# Revealing the neural representations underlying other-race face perception

**DOI:** 10.3389/fnhum.2025.1543840

**Published:** 2025-03-05

**Authors:** Moaz Shoura, Yong Z. Liang, Marco A. Sama, Arijit De, Adrian Nestor

**Affiliations:** Department of Psychology at Scarborough, University of Toronto, Toronto, ON, Canada

**Keywords:** EEG, other-race effect, image reconstruction, visual bias, face space

## Abstract

The other-race effect (ORE) refers to poorer recognition for faces of other races than one’s own. This study investigates the neural and representational basis of ORE in East Asian and White participants using behavioral measures, neural decoding, and image reconstruction based on electroencephalography (EEG) data. Our investigation identifies a reliable neural counterpart of ORE, with reduced decoding accuracy for other-race faces, and it relates this result to higher density of other-race face representations in face space. Then, we characterize the temporal dynamics and the prominence of ORE for individual variability at the neural level. Importantly, we use a data-driven image reconstruction approach to reveal visual biases underlying other-race face perception, including a tendency to perceive other-race faces as more typical, younger, and more expressive. These findings provide neural evidence for a classical account of ORE invoking face space compression for other-race faces. Further, they indicate that ORE involves not only reduced identity information but also broader, systematic distortions in visual representation with considerable cognitive and social implications.

## Introduction

The other-race effect (ORE), poorer recognition for faces of a different race than one’s own, has been consistently observed across an array of cultures and races (Hugenberg et al., 2010; Malpass and Kravitz, 1969). Given the scope of its real-world impact, ranging from failures of eye-witness testimony (Wells and Olson, 2001) and identity verification (Sporer et al., 2022) to difficulties with social interactions (McKone et al., 2023), ORE features prominently in the study of visual cognition (for reviews see Ficco et al., 2023; Kubota et al., 2012; Rossion and Michel, 2011). Hence, much has been learned about the relative contribution of perceptual, memory and social factors to its emergence as well as about the neural systems which they engage (Herzmann et al., 2018; Hughes et al., 2019; Natu et al., 2011; Wang et al., 2023; Zhou et al., 2020). Yet, much less is known about the neural representations underlying ORE, their visual content and intrinsic biases.

Accordingly, here, we aim to uncover visual representations involved in other-race (OR) versus same-race (SR) face perception with the aid of neural decoding and image reconstruction applied to electroencephalography (EEG) data (Nemrodov et al., 2018; Nestor et al., 2020). Specifically, we investigate the representational structure and the visual content responsible for ORE in East Asian and White participants. Further, we examine the prominence of ORE in neural face processing and its dynamics.

A seminal framework in the study of ORE is that of psychological face space, a multidimensional construct in which faces are represented as points and their pairwise distances correspond to their perceived similarity (Valentine, 1991). In this space, OR faces are often described as separate from SR ones (Jaquet et al., 2008) and more densely clustered (Byatt and Rhodes, 2004; Papesh and Goldinger, 2010). Such clustering accounts for the diminished discriminability of OR faces in experimental settings as well as for the real-world visual phenomenology (e.g., “to the uninitiated American, all Asiatics look alike, while to the Asiatic all white men look alike”; Feingold, 1914). The representational homogeneity of OR faces also finds ground at the neural level, where it elicits repetition suppression, as reported across multiple neuroimaging modalities (Hughes et al., 2019; Zhou et al., 2020). Specifically, viewing pairs of different-identity OR faces may elicit a reduction in neural response comparable to that induced by same-identity face pairs, suggesting that OR faces *look alike* even to the relevant neural population (Reggev et al., 2020; Vizioli et al., 2010).

The density of OR clusters highlights a reduction in facial identity information for OR representations and a tendency to collapse them to a prototypical face. However, the optimization of face space dimensions for SR representations (Valentine, 1991) also suggests that OR faces are encoded by suboptimal visual features corresponding to these dimensions (Dahl et al., 2014; O’Toole et al., 1991). Accordingly, a search for ORE-relevant features has led to extensive debates regarding the mis/use of facial shape and surface information (Balas et al., 2011; Balas and Nelson, 2010; Michel et al., 2013; Zhou et al., 2021), and the differential reliance on holistic/featural information (Harrison et al., 2014; Michel et al., 2006; Tanaka et al., 2004; Zhao et al., 2014). Yet, the misrepresentation of OR faces, beyond a reduction in identity information, remains to be elucidated. The present work addresses this challenge by investigating and leveraging the neural counterpart of ORE.

Neural sensitivity to ORE has previously been captured by event-related potentials (ERPs; Tüttenberg and Wiese, 2023), such as enhanced amplitude of the N170 component for OR faces (Senholzi and Ito, 2013; Walker et al., 2008; Wiese and Schweinberger, 2018); however, other studies reported no difference (Caldara et al., 2004; Tanaka and Pierce, 2009). Further, functional magnetic resonance imaging (fMRI) studies have reported higher activity in the fusiform face area (FFA) for SR than OR faces (Feng et al., 2011; Golby et al., 2001), though this difference may be more prominent for unfamiliar faces (Kim et al., 2006) and/or reflect a more general benefit for peer perception (Dai and Scherf, 2023). Relevantly here, multivariate fMRI analyses have revealed different spatial activation patterns in the ventral cortex for OR versus SR faces (Contreras et al., 2013; Natu et al., 2011; Wang et al., 2023) and have suggested different dynamics (Zhou et al., 2020).

Thus, while prior work highlights important aspects of OR perception, such as face-space clustering (Byatt and Rhodes, 2004; Papesh and Goldinger, 2010) and misrepresentation (Balas and Nelson, 2010; Zhou et al., 2021), their neural basis remains largely unexplored. Similarly, the full extent and the nature of OR face representations require broader investigation. Here, we attempt to shed light on these topics by: (i) relating neural decoding to behavioral ORE estimates; (ii) characterizing the temporal dynamics of OR versus SR perception, and (iii) retrieving the neural representations underlying ORE. To anticipate, our results show that: (i) ORE can be reliably related to differences in the neural decoding of OR versus SR faces; (ii) the neural dynamics of ORE evince an extensive time course, and (iii) OR face representations exhibit a typicality bias as well as age and expressiveness biases.

## Materials and methods

### Participants

A total of 43 adults (age range: 18–30 years; 28 females) from the University of Toronto community volunteered for the EEG experiment. After excluding three participants (2 East Asian, 1 White) due to technical difficulties with the EEG recordings, 20 identified themselves as East Asian and 20 as White. A majority (60%) of the remaining East Asian participants were international students from a Han Chinese background; in contrast, all White participants were locals (from Toronto, Ontario). All participants were right-handed, with normal or corrected-to-normal vision, and no history of neurocognitive disorders.

Prior work (Estudillo, 2021; McKone et al., 2012) on ORE with East Asian and White participants has estimated a medium effect size in both populations (Cohen’s *d* ≥ 0.62). A power analysis (JASP 0.17.1; jasp-stats.org) for an effect size of 0.60 (80% power; 5% Type I error rate) indicated that a sample size of 19 participants per group is needed. Thus, our final sample size should allow detecting ORE reliably.

Further, to validate behaviorally our EEG-based reconstruction results, we recruited online (Prolific; www.prolific.co) 50 additional adults (18–35 years; 23 female), referred to below as *validators*. Two were removed because of face recognition scores below the normative range (see below) and two due to failing reliability checks (i.e., negative correlation with themselves across repeated trials). Of the remaining validators, 23 were East Asian, born in East Asian countries, and 23 White, born in North America or Europe.

All participants/validators provided informed consent and received monetary compensation. All procedures were approved by the Research Ethics Board at the University of Toronto.

### Stimuli

For the EEG experiment we selected 30 images of East Asian males and 30 of White males of similar age, with frontal view, frontal gaze and neutral expression, from the Chicago Face Database (Ma et al., 2015). Images were standardized by: (1) aligning the position of the eyes and nose; (2) cropping to display only internal facial features; (3) normalizing with the same mean/contrast values in each CIEL*a*b* channel; and (4) resizing to 98 × 75 pixels. Resulting stimuli subtended a visual angle of 4° × 2.6°.

### Experimental procedures

First, participants completed two versions of the Cambridge Face Memory Test (CFMT) with Chinese (McKone et al., 2012) and White (Duchaine and Nakayama, 2006) face stimuli. This served to assess their face processing proficiency relative to the normative range (i.e., mean ± 2SD, as determined by prior work; Bowles et al., 2009; McKone et al., 2012) and, also, to behaviorally quantify ORE. Both tests have excellent psychometric properties (e.g., Cronbach’s *α* of 0.89–0.90; McKone et al., 2012; Wilmer et al., 2010) and can be used to estimate individual ORE via the traditional subtraction method (i.e., CFMT score for SR minus that for OR faces; Estudillo, 2021; Wan et al., 2015).

**Figure 1:**
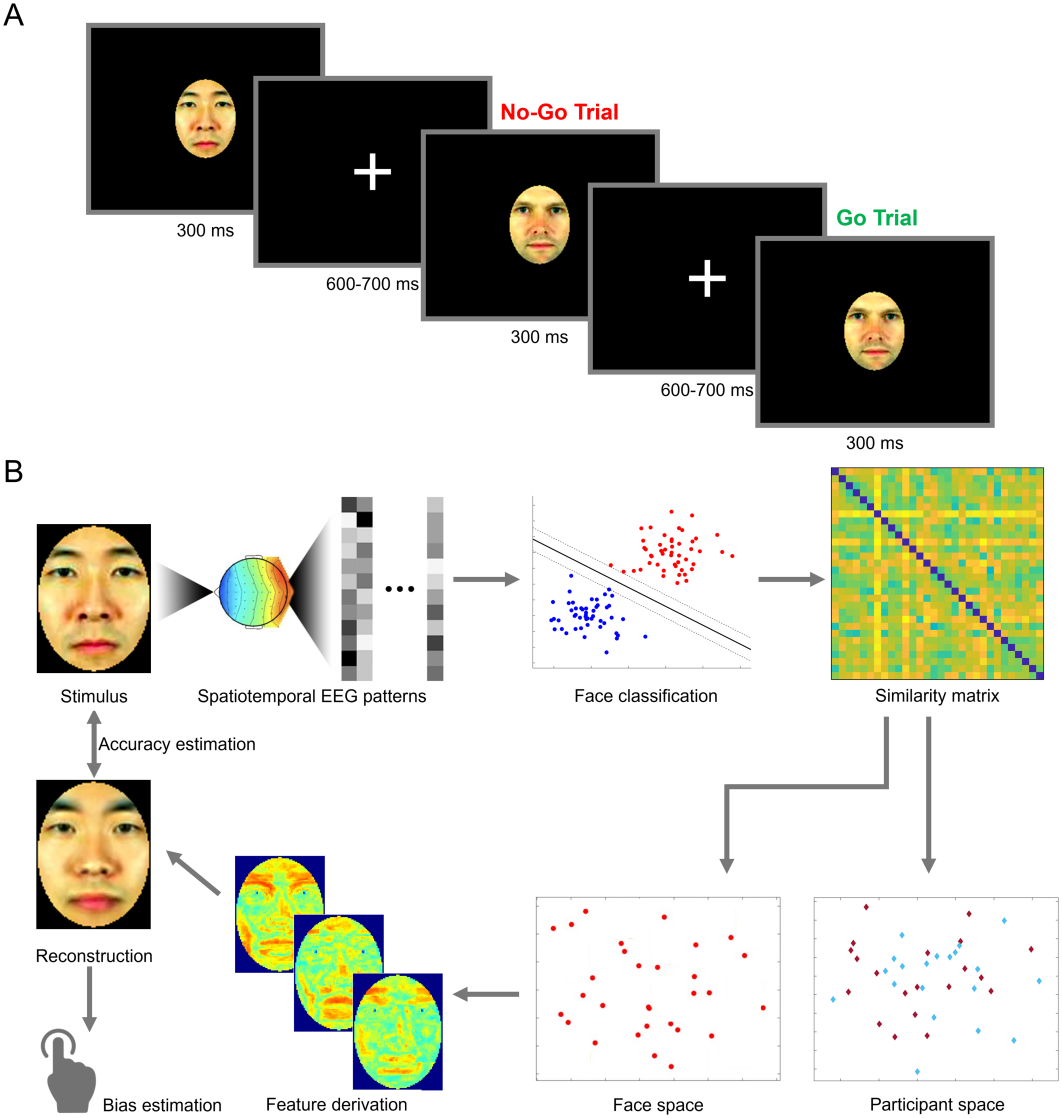
Schematic illustration of an experimental trial and of data analysis. **(A)** Participants performed a one-back go/no-go task in response to brief presentations of face stimuli. **(B)** Spatiotemporal electroencephalography patterns (EEG) elicited by viewing own- and other-race face stimuli (top left) are linearly decoded and converted into a representational similarity matrix (top right). Pairwise similarity ratings are then converted into EEG-based face space and participant space constructs (bottom right), through multidimensional scaling and principal component analysis, respectively. Facial features are derived directly from the structure of face space and combined into an image reconstruction aiming to recover the neural representation of the corresponding stimulus. The reconstruction is assessed in terms of image accuracy, with respect to its corresponding stimulus, and in terms of potential biases by human observers (bottom left). Facial images adapted from Ma et al. (2015).

EEG testing was conducted across two 2.5-h sessions on different days. Participants performed a go/no-go task by pressing a key upon the repetition of the same image on two consecutive trials. Data collection was divided across 32 blocks spanning the two sessions. Within any block, each stimulus was displayed four times, and a random 10% of trials featured a repetition (for a total of 264 trials/block). Stimuli were presented in pseudorandom order (e.g., to avoid repeated “go” trials). Each stimulus was displayed for 300 ms, followed by a variable 600–700 ms inter-stimulus interval during which a center fixation cross replaced the stimulus (see Figure 1A). Each session began with a practice block. Stimulus presentation and data collection relied on MATLAB and Psychtoolbox 3.0.8 (Brainard, 1997; Pelli, 1997). Both sessions were identical in structure, with stimuli presented on a 60 Hz, 1920 × 1080p display, from a distance of 80 cm.

### Analyses

#### Behavioral data analysis

Own-race CFMT scores were inspected to confirm that participants exhibit normal face recognition abilities. A two-way ANOVA (2 CFMT versions: East Asian, White; 2 participant groups: East Asian, White) was followed by within-group comparisons (paired *t*-tests) to confirm and compare ORE size.

To add confidence in significant findings and to aid the interpretation of statistically nonsignificant results, we employed Bayesian hypothesis testing (JASP 0.17.1) to report *BF*_10_ values, providing weight in favor of the alternative (*BF*_10_ > 1) or null (*BF*_10_ < 1) hypotheses. The analysis relied on standard default distributions for unspecified priors (e.g., uniform distribution for ANOVA analogs and Cauchy’s distribution for *t*-test analogs). We interpret results using established benchmarks (BF values in favor of alternative hypotheses: 1–3 anecdotal, 3–10 substantial, 10–30 strong, 30–100 very strong, > 100 extreme; and in favor of null hypothesis: 0.33–1 anecdotal, 0.10–0.33 substantial, 0.01–0.03 very strong, < 0.01 extreme; Wagenmakers et al., 2011).

#### EEG acquisition and preprocessing

EEG signals were recorded with a 64-channel Biosemi ActiveTwo system (Biosemi B.V.). Electrodes were configured according to the international 10/20 system. Offsets were maintained under a threshold of 40 mV. The signal was low-pass filtered using a fifth order sinc filter with a half-power cutoff at 204.8 Hz and, then, digitized at 512 Hz with 24 bits of resolution. All data were digitally filtered offline (zero-phase 24 dB/octave Butterworth filter) with a bandpass of 0.1–40 Hz and segmented into epochs extending from −100 to 900 ms. Epochs underwent direct current (DC) removal, linear detrending and baseline correction. Noisy electrodes were interpolated if necessary (no more than two electrodes per participant) and epochs were re-referenced to the average reference. Preprocessing relied on Letswave 6 (Mouraux and Iannetti, 2008) and Infomax ICA (Delorme et al., 2007) for artifact removal.

Relatively few trials contained false alarms as participants performed the task at ceiling (mean sensitivity d’ = 2.73). “Go” trials and those containing artifacts or false alarms were excluded, resulting in an average of 98.1% remaining trials across participants for further analysis.

#### Pattern classification analyses

We selected 12 electrodes positioned over occipitotemporal (OT) regions (P5, P7, P9, PO3, PO7, O1 on the left, and P6, P8, P10, PO4, PO8, O2 on the right). Their choice was based on their value in decoding facial information in the temporal domain (e.g., as previously determined by multivariate channel selection; Nemrodov et al., 2020) – for univariate results, see Supplementary materials and Supplementary Figure S1.

Consistent with this choice, decoding analyses relying on all electrodes yielded lower, though still significant, levels of classification accuracy compared to those relying on all electrodes (see Supplementary Figure S2).

With respect to the temporal information included in the analysis, two different approaches were employed. First, for temporally-cumulative analyses (Nemrodov et al., 2018; Roberts et al., 2019), we selected a large 50–650 ms time window and, then, concatenated ERP amplitude values across electrodes and time points resulting in 3684-feature patterns (i.e., 12 OT electrodes by 307 time points) for each trial. The length of this windows aimed to capture both early and late stages of face processing (Ghuman et al., 2014; Nemrodov et al., 2016; Roberts et al., 2019). Patterns corresponding to repetitions of the same stimulus within a block were averaged and normalized via z-scoring separately for each feature (i.e., for each combination of time point and OT channel). This yielded, for each participant, a total of 32 observations, one per block, for each stimulus.

Second, for time-resolved analyses (Dobs et al., 2019; Nemrodov et al., 2016), we considered smaller ~10 ms windows (i.e., five ~1.95 ms adjacent time bins). Decoding performance was estimated at each position, for a total of 508 intervals, by sliding this window one bin at a time between −100 and 700 ms.

Pairwise classification was then computed for each participant and stimulus pair, for a total of 1770 pairs across face identity and race. To this end, we used linear SVM (c = 1) with leave-one-block-out cross-validation (across 32 blocks from both experimental sessions). The procedure yielded decoding estimates (i.e., classification accuracy) for each pair of faces. These estimates were then separately assessed for: (1) SR faces (e.g., White faces for White participants), (2) OR faces (e.g., White faces for East Asian participants), and (3) between-race faces (i.e., White versus East Asian faces for both participant groups). Chance-level accuracy was derived by randomly shuffling stimulus labels 1,000 times and recomputing classification estimates. Average accuracy across participants was compared to permutation-based chance both for temporally-cumulative analyses (one-sample two-tailed *t*-tests, Bonferroni-corrected across comparisons) and time-resolved analyses (Wilcoxon signed-rank test, FDR-corrected across time points).

#### EEG-based face space and participant space

To visualize the representational space underlying race perception, we derived an EEG-based face space for each group of participants. To this end, decoding estimates derived from temporally-cumulative analyses for all pairs of faces were averaged across participants in each group and organized into a dissimilarity matrix. Then, metric multidimensional scaling (MDS) was applied to each matrix to derive a face space construct for each participant group (i.e., 40-dimension spaces accounting for at least 90% variance). We anticipated that faces in this space would be clearly separated by race and, also, that OR faces would cluster more tightly than SR ones. To this aim, we computed and compared average pairwise Euclidean distances between each pair of SR faces, each pair of OR faces and each pair of between-race faces separately for each participant group and corresponding space.

A complementary analysis sought to estimate a participant space and account for its structure. To this end, principal component analysis (PCA) was applied to EEG-based decoding accuracy vectors across all participants. Specifically, for each participant we considered a decoding performance vector consisting of the pairwise classification accuracies across all stimuli (i.e., a 1770-element vector yielded by all pairs of 60 stimuli). These vectors were then concatenated across all participants (i.e., into a 1770 × 40 matrix). Following the application of PCA to this matrix, the first two components of the resulting space were probed for their relationship with behavioral metrics: we assessed the contribution of participant race, facial recognition ability (as captured by SR CFMT scores) and ORE (captured by the difference between SR and OR CFMT scores; Estudillo, 2021; Wan et al., 2015) to these components. To this end, behaviorally derived scores were correlated with PC scores for each dimension.

#### Image reconstruction procedure

The reconstruction procedure was applied to neural data to recover and to visualize internal representation associated with each face, as well as to probe for potential visual misrepresentations associated with ORE. Our reconstruction approach follows earlier efforts (Nemrodov et al., 2018; Roberts et al., 2019), which leverage the face space framework to extract visual features and recover neural representations by harnessing the rich spatiotemporal information of EEG patterns (see Figure 1B). This approach involves a sequence of steps, adapted from prior work (for review see Nestor et al., 2020).

First, estimates of pairwise decoding accuracy for all faces of the same race were placed into a dissimilarity matrix and averaged across participants, separately for each group. To reconstruct a target face, this was removed from the data and the structure of the resulting matrix was used to estimate the representation of the target while avoiding dependency.

Second, metric multidimensional scaling (MDS) was applied to the dissimilarity matrix to derive a face space. A total of 20 dimensions were retained (accounting for over 90% variance separately for each group and stimulus race).

Third, for each dimension of face space, corresponding coefficients were normalized via *z*-scoring and a classification image (CI) was computed through reverse correlation (see Murray, 2011, for review). Specifically, we computed the average of all non-target images weighted by their coefficients on the dimension considered.

Fourth, significant CI information was assessed using a pixelwise test. To this end, images were shuffled relative to their coefficients on a given dimension and a new CI was derived (for a total of 1,000 permutations). Then, the value of each pixel in the original CI was compared to those at the same location in the permutation-based CIs. Significant pixels were identified, separately for each CIE L*a*b*color channel (permutation test, FDR-corrected across pixels, *q* < 0.1). Informative CIs/dimensions were determined based on the presence of at least 10 significant pixels in at least one color channel. All other CIs were eliminated from the procedure ensuring that only significant facial information was included in the reconstruction.

Fifth, the target face was projected into the existing face space. This was computed by Procrustes alignment of a space X containing all faces, including the target, to the space Y used for feature derivation (and not including the target). Then, the target was projected from X to Y using the alignment coefficients. This procedure ensures that the features of the target are not used in its reconstruction, thus avoiding dependency.

Finally, each significant CI was weighted by its coordinates in the corresponding dimension. Then, the resulting images were linearly combined across dimensions, effectively generating an image analog of a given location in face space.

The approach above was applied separately for each face (i.e., by treating each face as a reconstruction target). By considering decoding patterns for SR and OR faces, we aimed to recover both types of visual representations separately in East Asian and White participants.

Further, image reconstructions were also computed based on a theoretical observer. To this end, the approach above was applied to L2 pixelwise distances across stimuli (instead of neural-based dissimilarity estimates.) Since stimuli were matched in terms of contrast, pose, gaze, cropping, etc., this similarity space, though based on a low-level metric, should still reflect relative information about the visual appearance of different individuals. This analysis yielded comparable levels of reconstruction accuracy for the two races (90.57 and 92.18% for East Asian and White faces, respectively; Mann–Whitney U test across stimulus faces, *p* = 0.221, *BF*_10_ = 0.468). Importantly, this ensured that any potential differences in neural-based reconstruction could not be explained away by obvious differences in the image properties of the two stimulus races.

#### Image reconstruction evaluation

The accuracy of the image reconstruction for any particular face was assessed by determining the proportion of instances on which a reconstructed image was closer to its target stimulus than to any other face of the same race via a pixelwise L2 distance. To be clear, 100% reconstruction does not indicate a perfect replication of the corresponding stimulus, but only that the reconstruction was more similar to this stimulus than to any other stimulus of the same race. Then, accuracy was compared against chance (i.e., a one-sample test against 50%) and against each other for reconstructions of different stimulus races using a bootstrap test (10,000 iterations).

To rule out the possibility that any differences between OR and SR reconstructions simply reflect differences in image quality (e.g., due to image blur, pixel noise, spatial distortions) we computed estimates of each reconstructed image via two complementary metrics. Specifically, we appealed to a common reference-based metric, the structural similarity index (SSIM; Wang et al., 2004), as well as to a reference-free metric that approximates perceptual judgements, the blind/referenceless image spatial quality evaluator (BRISQUE; Mittal et al., 2012). OR and SR estimates were then compared to each other using a Wilcoxon signed-rank test separately for each metric and stimulus race.

Last, to assess local, low-level pictorial differences of SR versus OR face reconstructions, we subtracted corresponding images generated from the two groups (i.e., a reconstruction of a given face based on the data of White participants from a reconstruction of the same facial identity based on data from East Asian participants). Then, the outcomes were assessed with a pixelwise permutation-based test. Specifically, we randomly shuffled the labels of the participants across groups (i.e., East Asian, White), recomputed average dissimilarity matrices, reconstructed face images for each group and subtracted corresponding images generated from the two groups. The initial image differences were then compared with their permutation-based counterparts to identify pixels yielding values different from chance (two-tailed pixelwise permutation test, 1,000 permutations; FDR-corrected across pixels, separately for each color channel). This analysis was conducted for every facial identity as well as for the average of all facial identities of the same race.

#### Behavioral evaluation of reconstruction results

A different group of participants (*validators*) evaluated and compared the reconstruction results. First, validators completed the two versions of CFMT, with East Asian (McKone et al., 2012) and White face stimuli (Duchaine and Nakayama, 2006), to assess face processing abilities and ORE.

Then, they viewed 120 image reconstructions (i.e., 30 East Asian faces, reconstructed twice, from each group of EEG participants, and 30 White faces, also reconstructed twice). Since these stimuli are reconstructions of percepts elicited by stimuli in our EEG experiment, their appearance is standardized the same manner (e.g., with respect to size).

On each trial, validators viewed pairs of reconstructions of the same face identity (i.e., derived from East Asian versus White participant data) and selected the face which appeared younger, more expressive, or more typical of its race. Faces were displayed until a response was recorded, with self-paced breaks between blocks to minimize fatigue. Each pair of reconstructions was presented twice, by swapping the left/right position of the reconstructions on the screen. The experiment comprised 6 blocks (3 facial attributes × 2 stimulus races) of 60 trials (30 reconstructions of a given race presented twice). Block and trial order were randomized. Stimulus presentation and data collection relied on PsychoPy (Peirce et al., 2019). Validators completed testing during a 45-min online session.

After data collection, selection rates were averaged across trials separately by each stimulus race and facial trait for each validator. Selection rates were coded so that values above 50% indicate that reconstructions from OR participants (i.e., White face images reconstructed from East Asian participants, or vice versa) appear younger, more expressive, or more typical of their race. Conversely, a score below 50% indicate that validators judge reconstructions from their own racial group in this manner. Selection scores were then compared to chance (one-sample *t*-tests across validators against 50%, Bonferroni-corrected) and with each other (three-way mixed ANOVA; two validator groups: East Asian and White, two stimulus races, and three facial traits: age, expressiveness, typicality). Last, trait-specific selection rates, averaged across validators, were correlated with each other across all facial identities of the same race (e.g., age and expressiveness rates for East Asian faces) to examine whether judgments capture same/different underlying bias(es) across traits.

## Results

### Behavioral performance

Face recognition abilities were assessed using the CFMT with Chinese (McKone et al., 2012) and White (Duchaine and Nakayama, 2006) face stimuli across all participants. An analysis of recognition performance (two-way ANOVA; 2 test versions: Chinese, White × 2 participant groups: East Asian, White) revealed an effect of test version (*F*(1,38) = 10.42, *p* = 0.003, $\eta_{p}^{2}$ = 0.22, *BF*_10_ = 2.01) and participant race (*F*(1,38) = 10.90, *p* = 0.002, $\eta_{p}^{2}$ = 0.22, *BF*_10_ = 17.85), along with an interaction (*F*(1,38) = 38.1, *p* < 0.001, $\eta_{p}^{2}$ = 0.50, *BF*_10_ = 35.69). *Post hoc* tests revealed that, as expected, White participants outperformed East Asian participants on the CFMT-White (*t*(38) = 5.43, *p* < 0.001, *d* = 1.72); however, the two participant groups performed comparably on the CFMT-Chinese (*t*(38) = 0.66, *p* > 0.999). The latter result may be due to participant background, as ORE tends to be diminished in cities with a diverse multicultural population (Zhou et al., 2022) –all White participants were locals from a highly diverse, multicultural city (see Participants).

**Figure 2:**
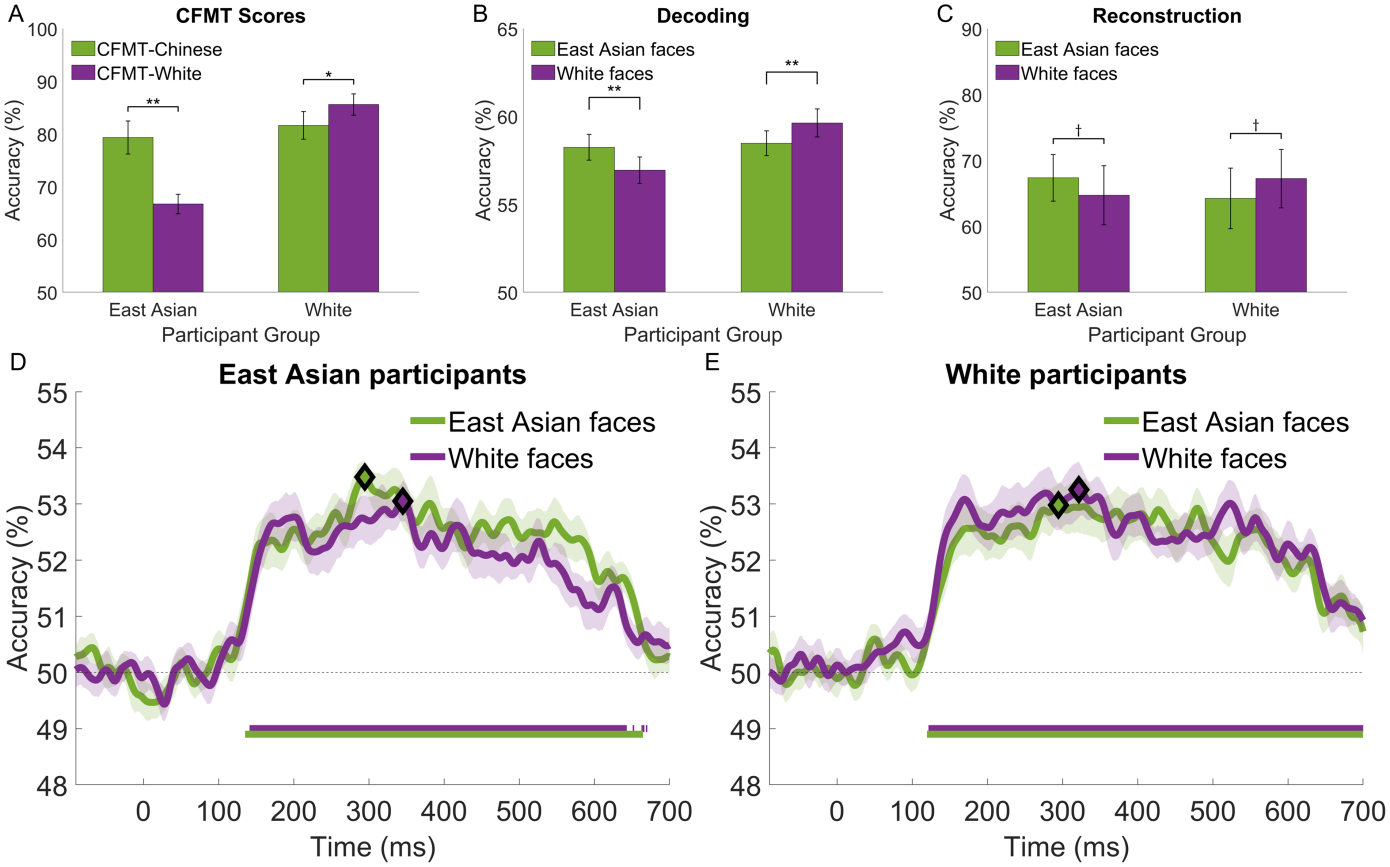
Performance associated with own- and other-race face perception for East Asian and White participants: **(A)** face recognition accuracy and other-race effects estimated with CFMT-Chinese and CFMT-White; **(B)** temporally-cumulative pairwise stimulus decoding by stimulus race (based on an 50-650 ms interval × 12 electrode patterns); **(C)** accuracy of EEG-based image reconstructions; **(D,E)** time-resolved face decoding (based on a 10 ms sliding window × 12 electrode patterns) for East Asian and White participants, respectively. Participants exhibit a systematic own-race advantage, which reaches significance (two-tailed *t*-tests across stimulus race) for **(A,B)**. The time course of face decoding shows extensive intervals of above-chance decoding (Wilcoxon signed-rank test against permutation-based chance; FDR-corrected across time points, *q* < 0.05; horizontal bars at the bottom of each plot) and higher estimates of own-race face decoding (though not significant after FRD correction). Diamonds mark the peaks of decoding accuracy in **(D,E)**. Error bars for **(A–C)** and shaded areas for **(D)**, **(E)** indicate ±1 SE (^†^ *p* < 0.10, *p < 0.05, ** *p*<. 01).

More importantly, ORE was confirmed by comparing SR to OR CFMT scores both for East Asian (two-tailed paired *t*-test, *t*(19) = 5.78, *p* < 0.001, *d* = 1.29, *BF*_10_ = 3178.03) and White participants (*t*(19) = 2.53, *p* = 0.02, *d* = 0.57, *BF*_10_ = 5.60)—see Figure 2A. Overall, these results confirm the presence of ORE in both groups, provide evidence for the robustness of our behavioral measures and motivate our investigation into their relationship with neural-based effects below.

### Neural decoding of SR and OR faces

Pairwise face decoding relying on temporally-cumulative decoding (Nemrodov et al., 2018; Roberts et al., 2019) revealed above-chance classification accuracy within and across stimulus race for both participant groups (one-sample *t*-tests across participants against permutation-based chance, all *p’*s < 0.001, all *d*s > 2.21, all *BF*_10_ values >3.71 × 10^6^) – see Figure 2B. An assessment of classification accuracy (two-way ANOVA; 2 stimulus races × 2 participant groups) revealed no main effects but a significant interaction (*F*(1,38) = 26.98, *p* < 0.001, η_p_^2^ = 0.42, *BF*_10_ = 36.1). Subsequent tests indicated that the decoding performance for East Asian faces was comparable in the two participant groups (*t*(38) = 0.22, *p* = 0.83). In contrast, White faces were marginally better decoded by White participants compared to East Asian participants (*t*(38) = 2.55, *p* = 0.058). We note that these results mirror the pattern of behavioral results described above, which we ascribe to participant background.

Importantly, a comparison of SR and OR face decoding revealed a significant advantage for the former both for East Asian (*t*(19) = 3.29, *p* = 0.004, *d* = 0.74, *BF*_10_ = 22.62) and White participants (*t*(19) = 4.51, *p* < 0.001, *d* = 1.0, *BF*_10_ = 245.31). Arguably, these results provide evidence for a neural-based ORE counterpart.

Further, between-race face decoding yielded higher decoding than within-race decoding both for East Asian participants (relative to White faces: *t*(19) = 8.41, *p* < 0.001, *d* = 1.88, *BF*_10_ = 3.71 × 10^5^ and East Asian faces: *t*(19) = 3.58, *p* = 0.002, *d* = 0.8, *BF*_10_ = 40.62) and for White participants (relative to White faces *t*(19) = 5.36, *p* < 0.001, d = 1.2, *BF*_10_ = 1405.80 and East Asian faces: *t*(19) = 6.85, *p* < 0.001, d = 1.53, *BF*_10_ = 2.40 × 10^4^).

We note that the present results are based on data from two experimental sessions for all participants. Separate session-specific analyses only yielded a significant SR advantage for the first but not the second session, potentially indicative of perceptual learning effects (see Supplementary Figure S3).

### Neural dynamics

To evaluate the temporal profile of face processing, decoding was conducted similarly over successive ~10-ms intervals (instead of a large window spanning most of the trial). For both participant groups and stimulus races, we note above-chance decoding (Wilcoxon signed-rank test against permutation-based chance; FDR-corrected, *q* < 0.05) starting around 130 ms, peaking between 300–350 ms and tapering off after 600 ms – see Figures 2D,E for East Asian and White participants, respectively.

Interestingly, for both groups, SR faces supported higher decoding accuracy across most time points after 130 ms, though not significantly after multiple-comparison correction (Wilcoxon signed-rank tests across stimulus race, *q* < 0.05). This suggests that the SR decoding advantage found by temporally-cumulative analysis is not due to any specific, restricted time window. Rather, it likely relies on aggregating complementary information over an extended interval. Accordingly, our results below capitalize on temporally-cumulative results rather than on temporally-restricted ones (e.g., such as those corresponding to decoding peaks).

### Neural-based face space

Accuracy estimates of pairwise face decoding, averaged across participants, were converted via multidimensional scaling (MDS) into a face space separately for each group (i.e., 40-dimension spaces accounting for at least 90% variance).

**Figure 3:**
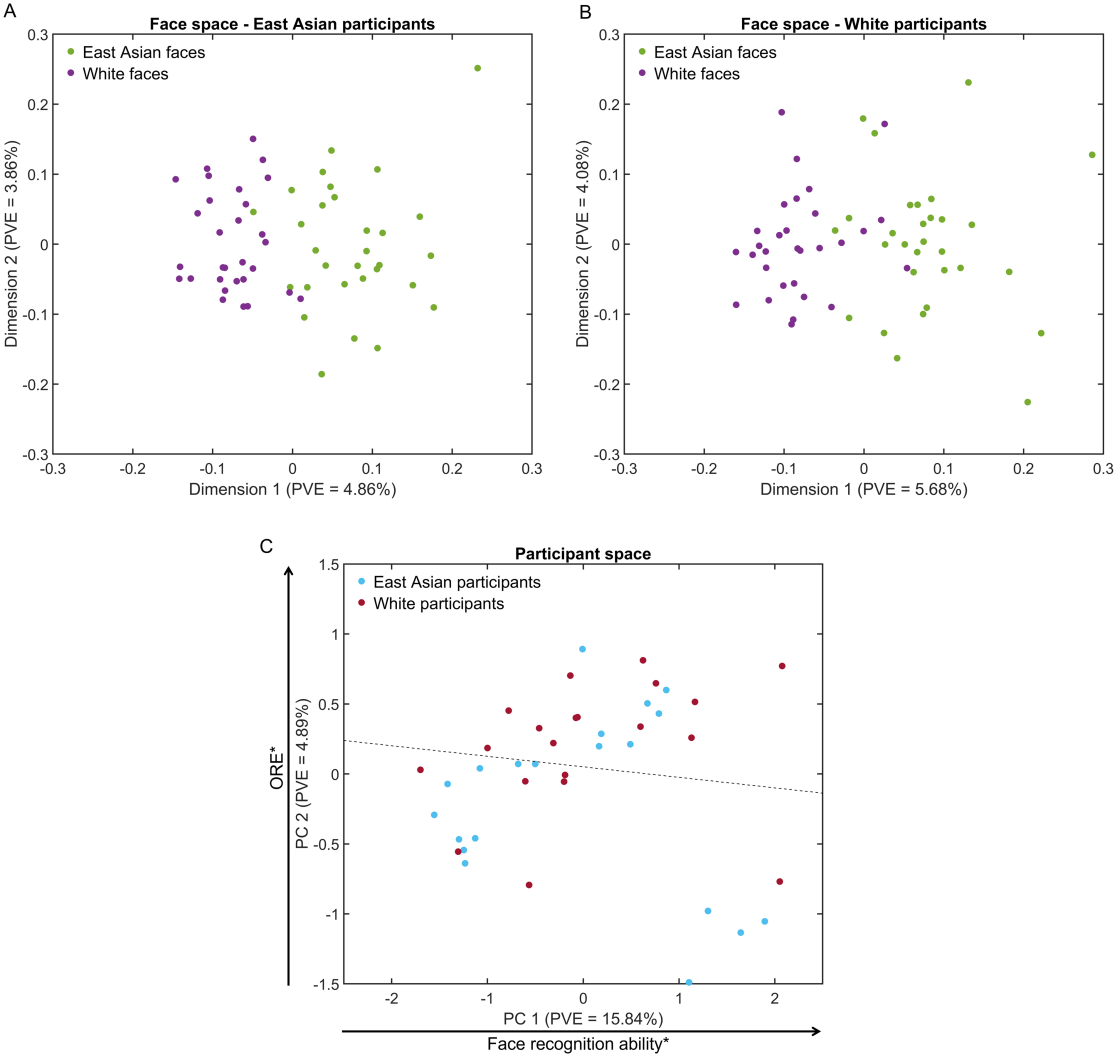
Representational spaces for face stimuli and participants. **(A,B)** Multidimensional face spaces for East Asian and White participants evince a clear separation of face representations by race and denser clusters for other-race faces (visible in two dimensions for **A**). **(C)** A participant space derived from EEG-based similarity vectors evinces some separation of participants by race (the dotted line marks a 65%-accurate hyperplane identified by logistic regression). Notably, participant scores across the first dimension are related to facial recognition ability (Pearson correlation with own-race CFMT across participants) while scores across the second dimension are related to other-race effect estimates (partial correlation with own-race minus other-race CFMT scores while controlling for participant race). PVE – percentage of variance explained (*p < 0.05).

The results evince a clear separation of faces by race (see Figures 3A,B). Additionally, we note a higher density for OR than SR faces, consistent with face space theory (Valentine, 1991) – in two dimensions this latter result is more clearly apparent for East Asian participants (Figure 3A). To assess this difference more rigorously, we computed pairwise face distances, using a Euclidean metric, across all dimensions. A comparison of such distances indicate that SR distances are, on average, 2.78% larger than OR ones for East Asian participants and 1.44% larger for White participants (Mann–Whitney U test, both *p*’s < 0.001, *BF*_10_ values >42.76) – see also Supplementary Figure S4 for heatmaps of all pairwise distances. Similarly, between-race distances are, on average, 2.36 and 4.34% larger than SR ones for East Asian and White participants (both *p*’s < 0.001, *BF*_10_ values >8303.45).

To confirm the robustness of the results above, we considered the potential impact of stimulus outliers. To this end, we identified stimuli whose distance from the center of their corresponding face group was larger than 2.5 SDs in face space, separately for each participant and stimulus race. This procedure identified one East Asian stimulus outlier for East Asian participants and one outlier per stimulus race for White participants. A comparison of SR, OR and between-race distances after outlier removal replicated the pattern of results above (all *p*’s < 0.001, all *BF*_10_ values >82.05).

These results reflect differences in decoding accuracy, as reported above, both within and across groups. Specifically, SR faces are better decoded than OR ones, leading to larger pairwise distances in face space for the former. At the same time, between-race faces are better decoded than within-race faces leading to even larger distances. Interestingly though, we note the prominence of race-based separation (i.e., along the first dimension of face space). These results, based on neural data, complement face space accounts of race informed by behavioral (Byatt and Rhodes, 2004; Papesh and Goldinger, 2010) and computational work (O’Toole et al., 1991; Caldara and Abdi, 2006; Wang et al., 2023).

To be clear, not all dimensions are likely to be (equally) informative regarding visual face representations and, thus, the estimates above can only serve as a coarse indication of ORE. Hence, a more careful examination of representational content and its sensitivity to ORE has to consider dimension/feature selection (e.g., as implemented by image reconstruction below).

### Relationship between neural decoding and behavioral performance

Behavioral and neural-based ORE estimates were computed for each participant via subtraction (i.e., SR-OR scores; Estudillo, 2021; Wan et al., 2015) from CFMT and decoding scores, respectively. These estimates were related to each other across participants from both groups (Pearson correlation, *r*(38) = 0.68, *p* < 0.001, *BF*_10_ = 1.30 × 10^4^). To assess whether this correlation reflected within-group differences, and not just a categorical difference across participant race, we conducted a partial correlation while controlling for participant race. This analysis yielded similar results (*r*(38) = 0.41, *p* = 0.009, *BF*_10_ = 5.53). The outcome highlights the relationship between behavioral and neural-based ORE.

Further, an exploratory analysis of EEG-based participant space, estimated via PCA from pairwise decoding results across participants, revealed some separation between the two participant groups, primarily along the second dimension. To visualize and quantify this separation, participant race was classified in PC space via logistic regression (i.e., across vectors of PCA coefficients corresponding to each participant). The classification reached an accuracy of 65% – see Figure 3C for the separating hyperplane.

To gain more insight into the structure of this space, as reflected by its first two dimensions, we assessed its relationship with estimates of facial recognition: own-race CFMT scores and ORE scores (i.e., SR – OR CFMT scores). We found that PC1 significantly correlated with SR CFMT scores (*r*(38) = 0.35, *p* = 0.029, *BF*_10_ = 1.98), while PC2 evinced a significant correlation with ORE scores (*r*(38) = 0.37, *p* = 0.02, *BF*_10_ = 2.70). Given that the stimuli and procedures used in our EEG experiment are considerably different from those used by the CFMT, these results demonstrate how race-related effects in face perception generalize across different stimulus sets and experimental procedures as well as across different data types. At the same time though, we acknowledge the need to confirm such correlational results using larger participant samples.

Thus, a neural-based participant space appears to be structured primarily by face recognition ability and ORE. Our present findings bolster the prominence of ORE at the neural level and provides ground for our investigation into the neural representations underlying ORE.

### Image reconstruction of SR and OR faces

EEG-based image reconstruction (Nemrodov et al., 2018; Nestor et al., 2020) recovered the visual content of SR and OR face representations. Above-chance reconstruction accuracy was found for both participant groups and stimulus races (two-tailed bootstrap test; all *p’*s < 0.001). Also, a trend for higher SR relative to OR accuracy (Figure 2C) was found for both East Asian (two-tailed bootstrap test; *p* = 0.062) and White participants (*p* = 0.065). We note that theoretical observer reconstructions supported equivalent levels of accuracy for the two races (see Image reconstruction procedure). Thus, any differences based on neural results are likely to stem from differences in the perceptual representations of our participants rather than, simply, from those in the low-level image properties of our stimuli.

Next, we considered that lower OR reconstruction accuracy may reflect poorer image quality relative to its SR counterpart (e.g., due to loss of high-frequency spatial information or to spatial distortions introduced by the reconstruction method). Accordingly, we evaluated image quality via a common reference-based metric (SSIM) of each reconstruction relative to its corresponding stimulus as well as via a reference-free metric (BRISQUE). A comparison of OR to SR reconstructions revealed no significant difference by either metric for faces of either race (Wilcoxon signed-rank test; all *p’s* > 0.42, all *BF*_10_ values <0.329, except for East Asian face reconstructions assessed with BRISQUE, *p* = 0.073, *BF*_10_ = 1.73). Hence, we conclude that any differences between OR and SR reconstructions do not reflect mere image quality differences (e.g., image blur).

**Figure 4:**
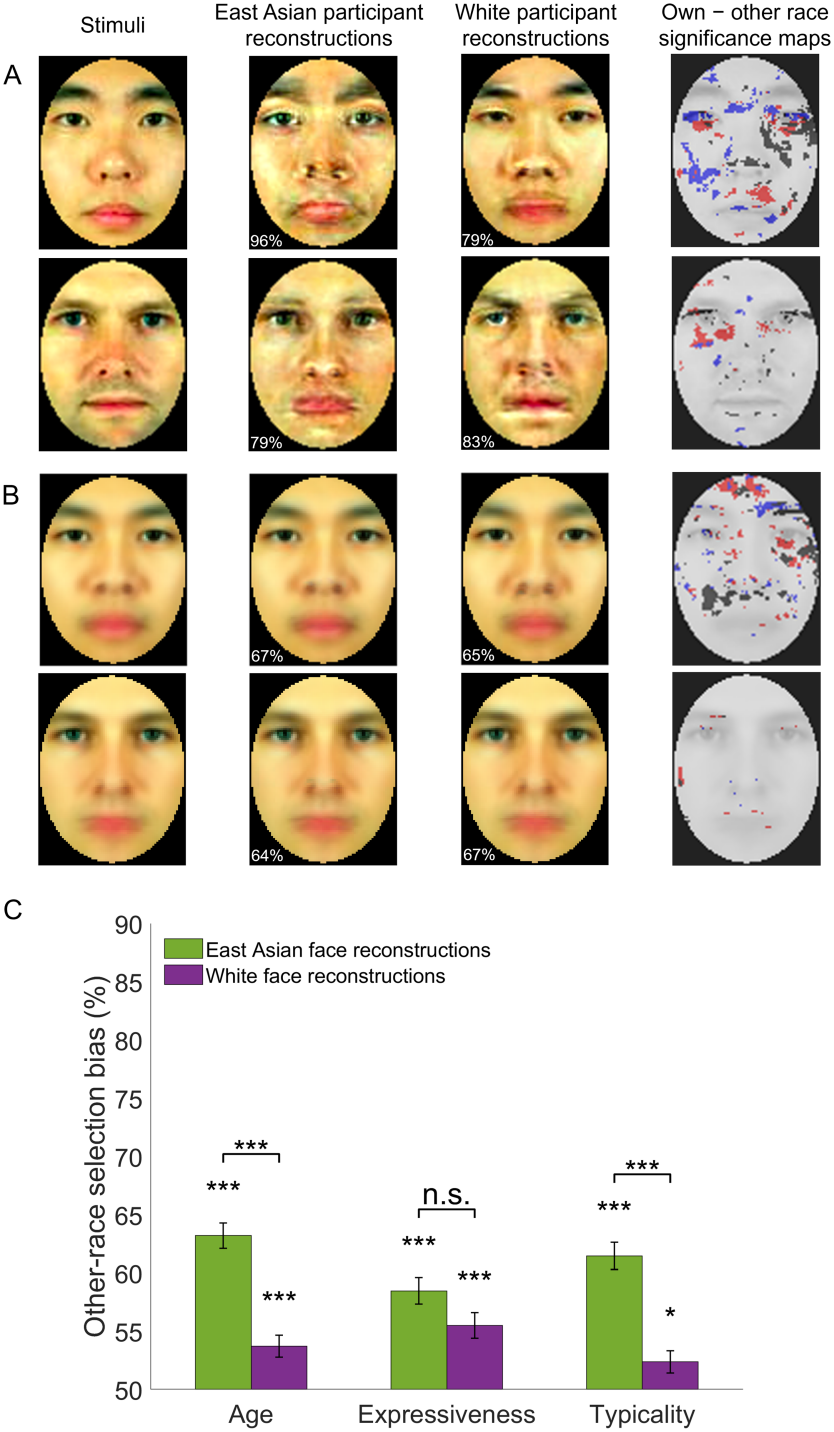
Own-race and other-race facial image reconstructions – examples and assessment. **(A)** Examples of individual face image reconstructions are shown for East Asian and White participants along with significance maps of the difference between own- versus other-race reconstructions (permutation-based pixelwise test, FDR-corrected, *q* < 0.05; dark/red/blue pixels mark areas yielding significantly different values in the luminance, red-green and yellow-blue CIEL*a*b* color channels). Numbers in the bottom left corner of each reconstruction indicate accuracy relative to the stimuli in the left column. **(B)** Averages of all reconstructions of the same race along with corresponding accuracy estimates and significance maps. **(C)** Other-race reconstructions are judged as younger, more expressive and more typical for their own race relative to own-race reconstructions of the same facial identities. Selection biases are significantly above chance (two-tailed *t*-tests against 50% chance in a 2-alternative forced choice task) and more pronounced for East Asian stimuli (paired *t*-tests for each facial trait). Error bars indicate ±1 SE (*p < 0.05, ***p < 0.001). Facial images adapted from Ma et al. (2015).

Further, to assess local, low-level pictorial differences between SR and OR reconstructions, a pixelwise permutation test was conducted, separately for each facial identity and color channel (two-tailed test, FDR corrected across pixels, *q* < 0.05). This revealed differences for all channels (Figure 4A) across multiple facial areas (e.g., around the eyes, eyebrows, nose). The analysis was repeated for image reconstructions averaged across all facial identities of each race (e.g., all Asian face reconstructions based on data from East Asian participants vs. those based on data from White participants) – see Figure 4B. However, the results are not immediately interpretable in terms of a systematic bias and somewhat less informative for White face reconstructions.

These results suggest that any divergence between SR versus OR image reconstructions may not be fully captured by low-level visual estimates. Accordingly, next, we asked a separate group of participants to assess the degree of race typicality for each of four sets of reconstructions (i.e., East Asian and White facial image reconstructions recovered from both groups of participants). In addition, we evaluated the possibility that OR visual representations are biased with respect to other facial traits, such as age (Dehon and Brédart, 2001; Mousavi et al., 2018) and expressiveness (Jiang et al., 2023; Yan et al., 2016), and asked validators to also judge these traits.

### Behavioral evaluation of image reconstructions

For each facial identity, validators viewed and compared its corresponding reconstructions derived from East Asian and White participants. Specifically, on separate trials, they selected the image in a pair that appeared younger, more expressive and more typical for its own race.

Average scores above 50% were noted for all three traits and both stimulus races (one-sample two-tailed *t*-tests, all *p’*s < 0.001, all *d*s > 0.58, Bonferroni-corrected, all *BF*_10_ values >109). These results indicate that, overall, OR faces are perceived as younger, more expressive, and more typical of their race (Figure 4C).

An assessment of selection scores (three-way mixed ANOVA; two validator groups: East Asian and White, two stimulus races, three facial traits) found no main effect of group (*F*(1,44) = 0.46, *p* = 0.50, *BF*_10_ = 0.232) or trait (*F*(2,88) = 1.84, *p* = 0.17, *BF*_10_ = 0.178) but a main effect of stimulus race (higher scores for East Asian than White faces: *F*(1,44) = 43.23, *p* < 0.001, η_p_^2^ = 0.50, *BF*_10_ = 3.54 × 10^5^) as well as an interaction between stimulus race and trait (*F*(2,88) = 7.39, *p* = 0.001, η_p_^2^ = 0.14, *BF*_10_ = 6.21 × 10^4^). Subsequent tests indicated that age and typicality scores were significantly more pronounced for East Asian than White face stimuli (*t*(44) = 6.13, *p* < 0.001, *d* = 1.30 and *t*(44) = 5.87, *p* < 0.001, *d* = 1.25, respectively) but not expressiveness (*t*(44) = 1.92, *p* = 0.23).

Next, trait-specific selection rates, averaged across participants, were correlated with each other across all facial identities of the same race (i.e., six correlations for each pair of traits and each stimulus race). All correlations were significant (all *p’s* < 0.001, all *BF*_10_ values >28.36, except for the correlation between expression and typicality of East Asian stimuli, *p* = 0.004, *BF*_10_ = 12.91; Bonferroni-corrected). To assess whether results were entirely driven by typicality, next, we computed partial correlations across age and expressiveness while controlling for typicality. Again, correlations reached significance for both stimulus races (both *p’s* < 0.001). Last, for completeness, these results were replicated after conducting the analysis separately for each group of validators, East Asian and White (both *p’s* < 0.001).

Overall, these results indicate that OR faces, regardless of participant race, are perceived as younger, more expressive, and more typical of their race. Last, these biases appear to reflect overlapping but not identical visual sources.

## Discussion

The present work investigates OR face perception with respect to its neural basis, its processing dynamics and its representational content. By relating neural decoding and image reconstruction results to behavioral performance in East Asian and White participants this investigation leads to several notable findings.

First, neural decoding, relying on temporally-cumulative occipitotemporal signals, mirrors the ORE evinced by behavioral performance. Specifically, between-race face decoding is more accurate than within-race decoding, consistent with the separability of neural patterns for OR from SR faces (Natu et al., 2011; Wang et al., 2023). More importantly, within-race face decoding is more accurate for SR than OR faces in both East Asian and White participants. This provides a decoding-based neural counterpart of ORE at the group level (i.e., across participant race), complementing results based on repetition suppression (Hughes et al., 2019; Vizioli et al., 2010; Zhou et al., 2020). Yet, behaviorally, ORE is also known to exhibit a gradient across individuals from the same group (e.g., Megreya et al., 2011). Hence, here, we also examine ORE’s prominence in neural processing at the individual level. This investigation points to ORE and face recognition ability as main components of variability in representational similarity across participants. Thus, our findings speak to the neural basis of ORE, both across and within groups, while also serving as a platform for the rest of our investigation.

Second, the temporal profile of decoding exhibits an extensive window of significance, between around 130–600 ms, consistent with prior work on the neural dynamics of facial identity perception (Ambrus et al., 2019; Dobs et al., 2019; Kovács et al., 2023; Nemrodov et al., 2018). Overall, this profile is similar for SR and OR faces as no specific time points, in isolation, yield a significant difference. However, an SR decoding advantage is, at least, numerically apparent during most of the 130–600 ms interval (Figures 2D,E). This suggests the presence of complementary information over time, which accrues into an overall SR advantage, captured by temporally-cumulative analysis – to be clear, the latter analysis concatenates, rather than averages, temporal information, thus allowing decoding to exploit temporal patterns over this extended window. While an ORE univariate effect for the N170 component was apparent for East Asian participants (see Supplementary materials and Supplementary Figure S1), our decoding results indicate that ORE is unlikely to be solely linked to a restricted temporal window. The interpretation above, of complementary information over time, also agrees with evidence for multiple stages of neural processing associated with ORE across an extensive cortical network (Natu et al., 2011; Zhou et al., 2020).

Third, face spaces derived from EEG data evince meaningful, informative structure. Previous fMRI (Loffler et al., 2005; Nestor et al., 2016; Carlin and Kriegeskorte, 2017) and neurophysiology (Chang et al., 2017) studies have yielded evidence for a neural-based face space whose dimensions encode visual features relevant for identity recognition. Our results agree with this prior work, though important aspects of these spaces, such as specific nonlinearities (Carlin and Kriegeskorte, 2017; Yang et al., 2023), remain to be examined for their EEG-derived counterparts here. Our investigation goes, however, beyond the ability of such spaces to encode individual appearance. Specifically, we focus on the separability of faces by race and the higher representational density of OR versus SR.

Relevantly here, prior behavioral (Tanaka et al., 2013; Papesh and Goldinger, 2010) and computational work (O’Toole et al., 1991) has pointed to OR compression in face space. For instance, Caldara and Abdi (2006) trained an autoassociative network only with White or only with Asian face images. In both cases, the authors found higher density for the left-out race in the face space projections of the network. Namely, they found larger pairwise distances, on average, for SR faces. Our results provide a neural counterpart of these simulations based on pairwise face discriminability. Overall, such findings suggest that visual experience with one race amplifies SR face dissimilarity by optimizing face space structure for SR face encoding and recognition (Valentine, 1991).

Fourth, we demonstrate the richness of visual information present in face space by successfully deploying its dimensions and associated features in image reconstruction. Accordingly, face representations, recovered through reconstruction, reveal visual differences between SR and OR faces which do not reflect mere differences in image quality. Specifically, image reconstructions assessed by a separate group of validators, revealed that OR faces are represented as more typical for their race. This agrees with OR compression in face space, as noted above, as well as with ORE phenomenology (i.e., “they all look the same”; Ackerman et al., 2006; Feingold, 1914; Laurence et al., 2016). Notably though, we also find largely separate biases in the perception of age and expressiveness, which we address next.

Little is known about OR biases in age perception, with a handful of prior studies yielding conflicting results (Dehon and Brédart, 2001; Mousavi et al., 2018; Zhao and Bentin, 2008). Recent behavioral work suggests that OR faces are represented as younger in both East Asian and White participants (Shoura et al., 2024). Further, an *illusion* of Asian youthfulness is suggested by inter-ethnic differences in both skin physiology (e.g., more collagen) and skeletal structure (Shirakabe et al., 2003; Vashi et al., 2016) relative to White faces. Therefore, we reasoned that OR faces may be perceived as younger than SR ones, at least by White participants. Our results support the hypothesis above as well as a reciprocal bias, with Asians also perceiving White faces as younger. Thus, the present findings speak to a general OR bias in age estimation and, critically, they reveal the visual representations supporting prior reports of such biases (Dehon and Brédart, 2001; Mousavi et al., 2018).

Regarding expressiveness, recent work has found poorer performance in OR expression recognition (Jiang et al., 2023; Yan et al., 2016), possibly due to cultural differences in the representation of emotional expressions (Chen et al., 2024; Jack et al., 2012). While our stimuli display neutral expressions, emotion can be perceived even in neutral faces as a function of facial structure (Neth and Martinez, 2009; Said et al., 2009) and person knowledge (Suess et al., 2015). Hence, we reasoned that biases in expression recognition may also extend to the degree of expressiveness perceived in OR neutral faces. The results bore out this hypothesis and, again, they revealed the representations underlying this bias. Further work though will be needed to uncover the precise nature of this bias (e.g., as driven by valence, arousal).

From a practical standpoint, we note that perceptual biases have critical implications for eyewitness testimony. For instance, a typicality bias, whereby OR faces are perceived as less distinctive, may exacerbate misidentifications in legal settings (Wells and Olson, 2001; Meissner and Brigham, 2001), even when confidence is high (Dodson and Dobolyi, 2016). Additionally, biases in expressiveness can influence judgments about emotional intent, introducing another layer of distortion in face recognition (Jiang et al., 2023). Prior research has evaluated training programs emphasizing individuation strategies, which may help mitigate such perceptual biases, enhance cross-racial identification and improve the reliability of eyewitness testimony (Young et al., 2012; Lebrecht et al., 2009).

While our examination of potential biases was driven by specific hypotheses, current results may be further queried for other facial trait biases (e.g., in attractiveness, competence). Methodologically, this opportunity illustrates the benefit of data-driven approaches aimed at recovering internal representations (Chen et al., 2024; Nestor et al., 2016, 2020; Zhan et al., 2019), including their ability to uncover new perceptual biases. Further, theoretically, it showcases how encoding OR faces in a suboptimal face space (Dahl et al., 2014; O’Toole et al., 1991), crafted for SR recognition, may lead to an array of representational distortions impacting multiple facial traits. In turn, practically, such an array of biases can shed new light on an ORE-induced decrement in the quality of social interactions (McKone et al., 2023), beyond difficulties with person identification.

Regarding the nature of the information underlying ORE, a qualitative advantage for SR versus OR emerged early, around 130 ms, consistent with the grounding of ORE in perception (Megreya et al., 2011). However, this advantage, exploited by our temporally-cumulative analyses, extended up to ~600 ms. Thus, these results do not allow linking ORE solely to an early, restricted temporal window associated with automatic perceptual processing. Prior work has argued for the contribution of memory (Herzmann et al., 2022; Tanaka et al., 2004; Yaros et al., 2019; Zhao et al., 2014) and socio-cognitive factors (Hugenberg et al., 2010; Schwartz et al., 2023) to ORE. Accordingly, we cannot rule out that such factors may have contributed, in addition to perceptual ones, to the visual representations assessed here.

Furthermore, we note that the main source of ORE may well vary across different groups. For instance, it may be driven by perceptual experience for Asian-White groups but by social-motivational factors across Black-White groups (Wan et al., 2015). Accordingly, investigating the nature and extent of visual biases across other groups (e.g., Black-White) could be very informative in this respect. Further, evaluating memory-based representations (Chang et al., 2017; Zhan et al., 2019) and their ORE biases could help clarify the interplay between perception and memory.

To conclude, the present work integrates measures of behavioral performance, neural decoding and image reconstruction to yield new insights into the representational basis of ORE and its dynamics. Our findings reveal multiple biases in OR face perception with significant theoretical, methodological and practical implications. More generally, they open new avenues for exploring racial biases in face recognition and pave the way to studying visual misrepresentations via image reconstruction.

## Data Availability

The datasets presented in this study can be found in online repositories. The names of the repository/repositories and accession number(s) can be found at: open science framework: https://osf.io/u3rsh/.
