# Supplementary material for: Revealing the neural representations underlying other-race face perception
Source: Front Hum Neurosci. 2025 Mar 5;19:1543840. doi: 10.3389/fnhum.2025.1543840 (PMC11920127; doi:10.3389/fnhum.2025.1543840)
Supplement: Supplementary file 1 [file Data_Sheet_1.docx]

**Supplementary Materials**

**Univariate analysis**

Extensive research and debate have targeted the sensitivity of the N170 ERP component to the other-race effect (ORE), with multiple studies reporting larger amplitudes for OR than SR faces (Balas & Nelson, 2010; Walker et al., 2008; Wiese & Schweinberger, 2018). However, other studies reported no differences (Caldara et al., 2004; Tanaka & Pierce, 2009) or task-dependent effects, such as larger OR amplitudes when participants attend to facial identity but lower when they attend to race (Senholzi & Ito, 2013).

Accordingly, here, we also assessed the modulation of N170 amplitude and latency by ORE. To this end, average ERP signals were separately computed for 6 left and 6 right occipitotemporal (OT) electrodes (see Methods, Pattern classification analyses), which evince a robust N170 component. The data were further averaged across different face stimuli by race. Three participants (one East Asian, two White) who yielded outlier values for N170 amplitude and/or latency (i.e., beyond ±2.5 SD from the mean of the other participants in their group) were excluded from further analyses. A three-way mixed-design ANOVA (JASP 0.17.1; jasp-stats.org) was conducted across remaining participants separately for amplitude and latency estimates, with participant group (East Asian, White) as a between-subjects factor, and stimulus race (East Asian, White) and lateralization (left, right electrodes) as within-subjects factors.

The analysis of N170 amplitudes revealed a significant main effect of stimulus race (larger amplitude for White faces relative to East Asian ones; *F*(1,35) = 6.54, *p* = .015, η_p_^2^ = .16, *BF*_10_ = 2.7) but no significant effects of participant group, lateralization or interactions (all *p*’s > 0.1). However, further examination revealed that the effect of stimulus race was present for East Asian (*t*(18) = 2.39, *p* =.03, *d* = .55, *BF*_10_ = 2.23) but not for White participants (*t*(17)= 1.07, *p* = .30, *d* = .25, *BF*_10_ = .39). Specifically, the N170 component evinces lower amplitude for OR faces in one participant group. While this effect is not significant in our White group, this may be due to the diminished behavioral ORE noted for this group (see Results, Behavioral performance). No main effects or interactions were found significant for a similar analysis of latency values (all *p*’s > .05). These results are largely consistent with prior work and, also, suggest that participants attend to facial identity rather than to facial race (Senholzi & Ito, 2013), accounting for our ability to discriminate within-race faces via neural decoding.

We note that several other ERP components (e.g., P1, P2, N250) also carry relevance for the study of ORE (for a recent review see Tüttenberg & Wiese, 2023). However, a thorough assessment of their univariate sensitivity to ORE is beyond the main goals of the present work and was not examined further.

**
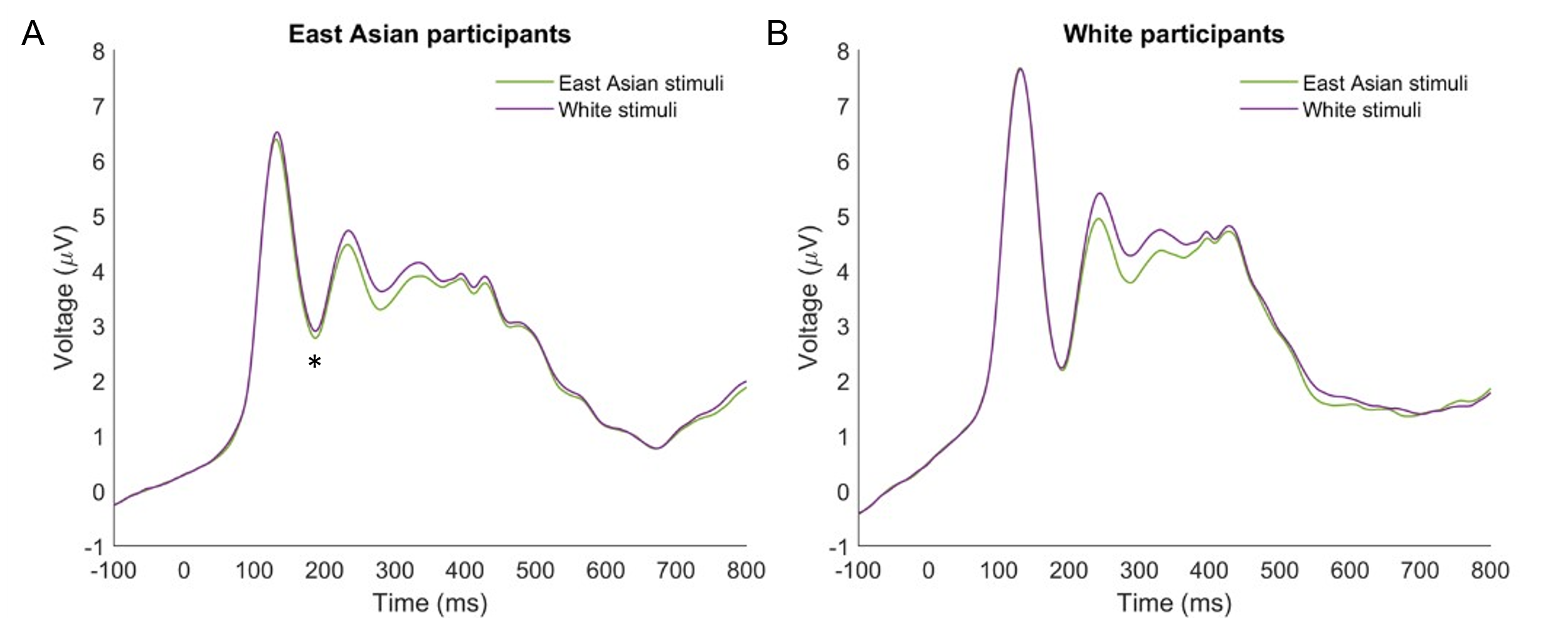
**

**Fig. S1.** ERP waveforms for East Asian participants (left) and White participants (right). The N170 component evinced higher amplitude for OR than SR faces in East Asian participants but not in White participants.


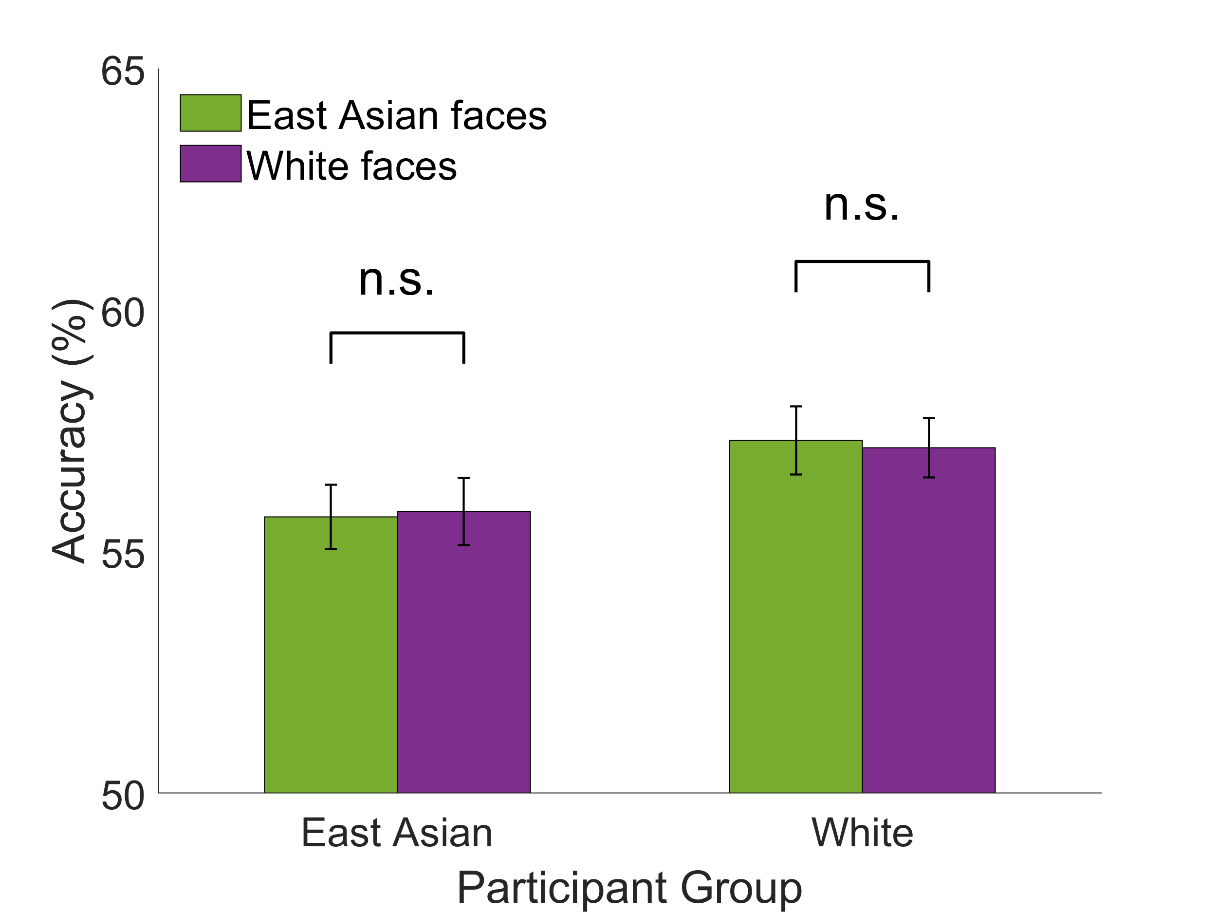


**Fig. S2.** Temporally-cumulative stimulus decoding by stimulus race and participant group. Decoding relies on a 50-650ms interval after stimulus onset and all 64 electrodes. Decoding performance is poorer relative to that based on 12 OT electrodes (Fig. 2B) and does not evince an own-race advantage (two-tailed t-tests across stimulus race for each participant group, both *p*’s > .10).


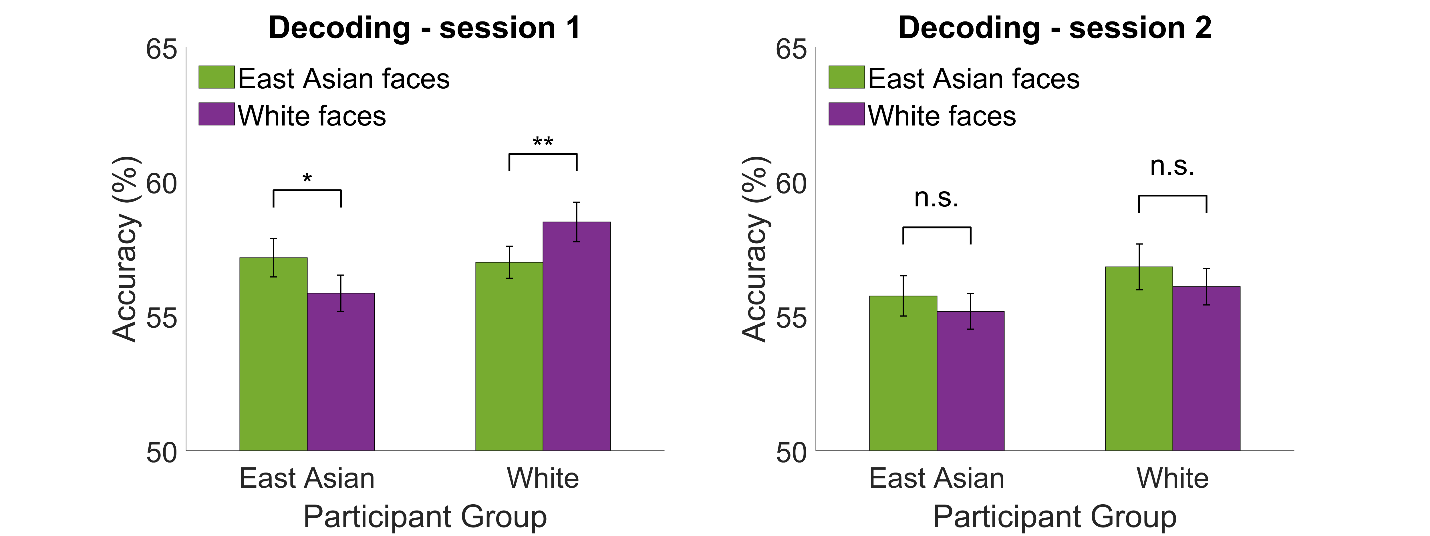


**Fig. S3.** Temporally-cumulative stimulus decoding by stimulus race and participant group, separately for (A) session 1 and (B) session 2. An own-race advantage is present for each participant group only in session 1 (two-tailed t-tests across stimulus race for each participant group (**p* <. 05, ***p* < .01).


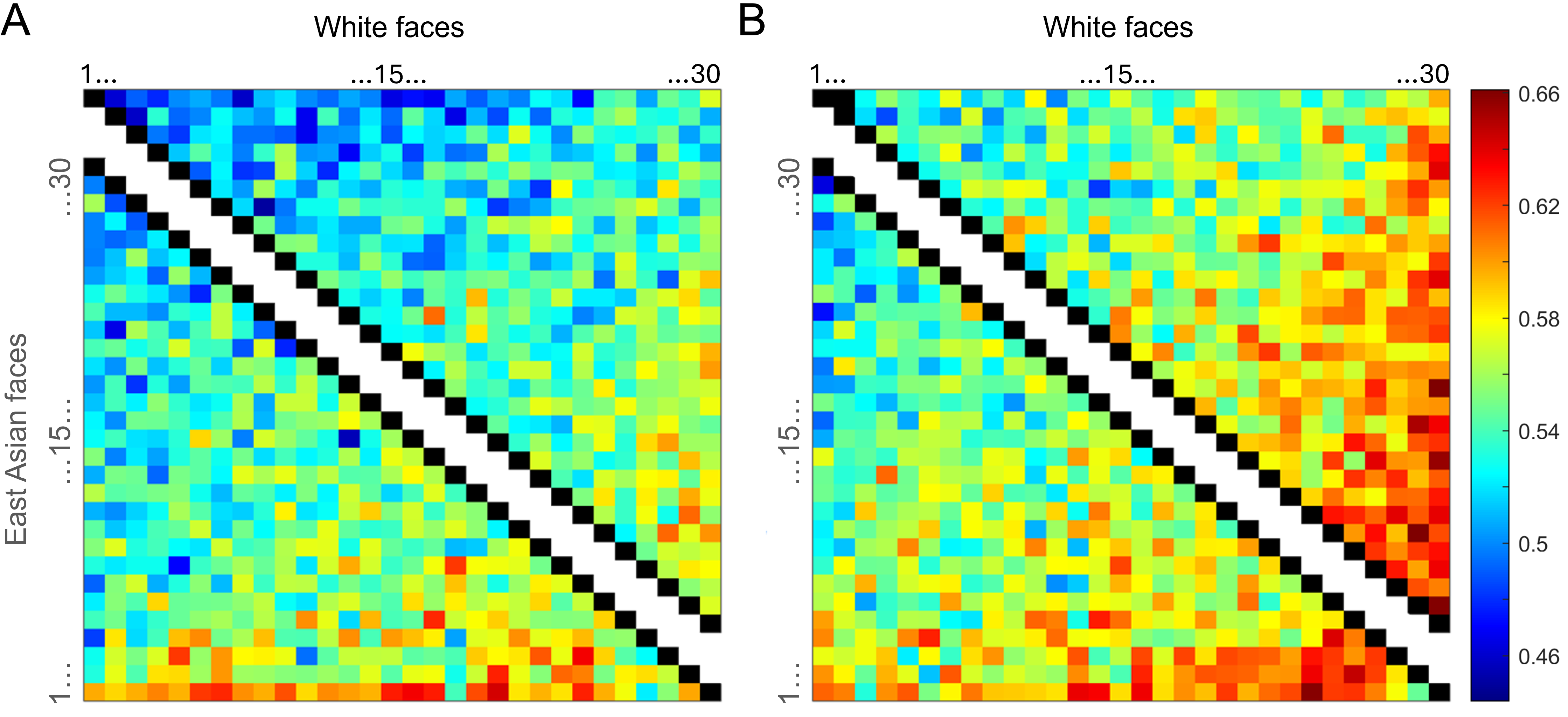


**Fig. S4.** Pairwise face space distances for (A) East Asian and (B) White participants. Each cell indicates the Euclidean distance (in arbitrary units) for pairs of East Asian faces (lower triangles) or White faces (upper triangles). Faces are ranked in ascending order, separately by stimulus race, from the most typical (i.e., closest, on average, to all other faces of the same race) to the most distinctive (i.e., the farthest from all other faces of the same race). SR faces, corresponding to the lower triangle in (A) and upper triangle in (B), evince larger average values relative to their OR counterparts (Mann-Whitney U tests, both *p*’s < .001).

**References**

1. Walker, P. M., Silvert, L., Hewstone, M. & Nobre, A. C. Social contact and other-race face processing in the human brain. *Soc. Cogn. Affect. Neurosci.* **3**, 16–25 (2008).

2. Balas, B. & Nelson, C. A. The role of face shape and pigmentation in other-race face perception: An electrophysiological study. *Neuropsychologia* **48**, 498–506 (2010).

3. Wiese, H. & Schweinberger, S. R. Inequality between biases in face memory: Event-related potentials reveal dissociable neural correlates of own-race and own-gender biases. *Cortex* **101**, 119–135 (2018).

4. Caldara, R., Rossion, B., Bovet, P. & Hauert, C. A. Event-related potentials and time course of the ‘other-race’ face classification advantage. *Neuroreport* **15**, 905–910 (2004).

5. Tanaka, J. W. & Pierce, L. J. The neural plasticity of other-race face recognition. *Cogn. Affect. Behav. Neurosci.* **9**, 122–131 (2009).

6. Senholzi, K. B. & Ito, T. A. Structural face encoding: How task affects the N170’s sensitivity to race. *Soc. Cogn. Affect. Neurosci.* **8**, 937–942 (2013).

7. Tüttenberg, S. C. & Wiese, H. Event-related brain potential correlates of the other-race effect: A review. *Br. J. Psychol.* **114**, 24–44 (2023).
